# Retained Surgical Drains in Orthopedics: Two Case Reports and a Review of the Literature

**DOI:** 10.1155/2017/8194571

**Published:** 2017-04-19

**Authors:** John S. Cox, Darin Friess

**Affiliations:** Department of Orthopaedics and Rehabilitation, Oregon Health & Science University, 3181 SW Sam Jackson Park Rd., Mail Code OP31, Portland, OR 97239, USA

## Abstract

Though a relatively rare event, retained surgical drains are preventable and can lead to significant consequences. Two case reports from our institution are discussed as examples for the challenging management of this problem as well as an overview of techniques for the prevention and removal of retained drains based on the current literature.

## 1. Background

Retained surgical drains are an uncommon yet entirely preventable complication. There are several reports of retained surgical drains in the orthopedic literature, as well as in many other subspecialties that regularly employ the use of drains [1–3]. Most commonly this problem occurs when suturing the drain in at the time of wound closure or through drain breakage [4]. The aim of this review is to inform surgical teams about techniques to avoid suturing-in drains, as well as techniques to remove the retained drain potentially without returning to the operating room.

### 1.1. Case 1

**Figure 1:**
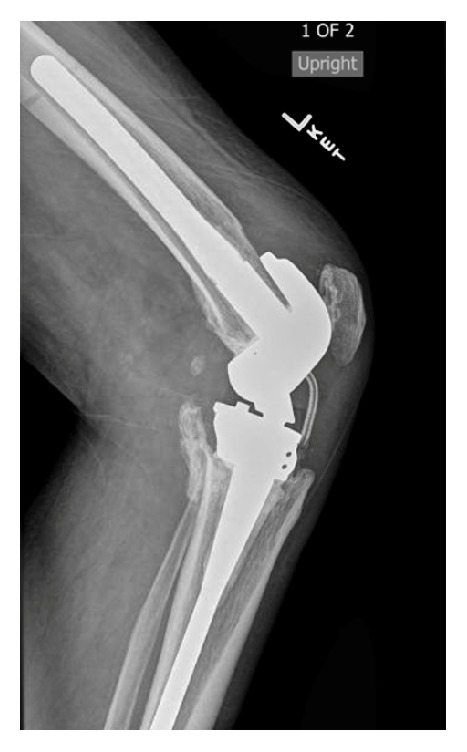
Note retained drain deep to patellar tendon.

An 82-year-old male presented with a Schatzker Type 6 tibial plateau fracture after being struck by a motor vehicle. The patient was treated in a staged fashion with initial external fixation, followed by a subsequent complex total knee replacement (TKR) using a hinged prosthesis. A Hemovac™ drain was placed at the time of procedure and an attempt was made to remove it on the first postoperative day which was met with great resistance. When the drain finally yielded, it was found to be broken. This was confirmed with radiographs (Figure 1).

The patient initially decided against removing the drain as he did not want to undergo another procedure. His postoperative course was complicated by a flexion contracture and the feeling of a foreign object in the knee. After struggling for five months with these issues, he decided to have the drain removed. At the time of the operation, the retained drain was removed and his knee regained full extension with manipulation under anesthesia.

### 1.2. Case 2

A 35-year-old male suffered an anterior-posterior compression pelvic injury following a crush injury from a horse. He underwent open reduction internal fixation of his pubic symphysis and percutaneous fixation of his right sacroiliac joint. A Jackson-Pratt™ drain was placed in the Space of Retzius prior to closure. The surgical resident attempted to remove the drain on the second postoperative day. The drain had significant resistance. Because of associated pain a second attempt was made under oral analgesia. However, the drain broke at the interface between the clear tubing and the white fenestrated portion. Finally the remaining part of the drain had to be removed under general anesthesia. The remaining drain was found to have no punctures or other signs of inadvertently being sutured-in. The surgical team's impression was that a tight fascial closure did not accommodate the wider drain portion causing failure at the tube-drain interface. The patient's remaining course was uneventful and he healed without complication at the last follow-up 5 months postoperatively. If something is to be learned from this, it would be to routinely place a portion of the widest part of the drain above the fascia to ensure that the fascial closure will accommodate the widest drain portion. This is the first known reported failure of the drain at the tube-drain interface.

## 2. Techniques

There are several techniques described for the prevention, confirmation, and management of retained surgical drains.

### 2.1. Confirmation Techniques

Traditional surgical education is the basis of this technique and involves the near-universal practice of cutting the drain between the drain holes [5]. If the drain were to break, the break would occur at the weak point through the perforations. This would be evident at the time of removal and would suggest there is a retained portion.

Jaafar et al. [5] recommended purposefully cutting the drain in order to have a consistent number of holes each time. The number of holes should be counted at the time of removal to be assured there are no retained fragments. This is described for total joint arthroplasty, as these surgeries are fairly consistent in the wound size and the number of drain holes. In cases in which the length of drain is variable, the number of holes left in the drain should be documented in dictation and confirmed at the time of removal.

### 2.2. Prevention Techniques

The first technique involves leaving slack in the drain such that the black dot or another marker (the mark on the drain denoting the appropriate skin level) is buried below the skin. Tuck the drain into the lateral gutter for TKR or below the iliotibial band for Total Hip Replacement. After closing, pull the slack out until the marker is at the skin. If it glides easily, it is unlikely to be sutured-in [5].

Another technique recommends, after passing the trocar, keeping the free end of the drain long and protruding 2-3 cm from the distal end of the wound. After fascial or arthrotomy closure, pull the proximal aspect (trocar end) until the protruding end slides under the closed layer. Any resistance indicates that the drain is tethered [6, 7].

A final technique involves placing a hemostat on the free end of the drain. Once the layer above the drain is closed, use a sliding motion to and fro to evaluate for any resistance before proceeding with the closure [8].

### 2.3. Removal Techniques

If applying a firm pull fails in routine drain removal, the following techniques are available to try at the discretion of the operating surgeon and tolerance of the patient. If there is concern that a portion of the drain broke off within the wound, radiographs should be obtained to confirm as most drains have slight radiopacity.

Redman et al. [9] described a technique for removal of entrapped Penrose drains. This can be applied to other drain types as well. Apply gentle traction using one hemostat, while a second hemostat is advanced along the drain as far as possible and clamped. Remove the first hemostat and apply gentle traction with the second clamp. Continue this in an alternating fashion until the suture is encountered, at which time it can be cut. This technique was successful in 4/6 cases in dogs. However, they found that if the suture is >7 cm deep, this technique will not likely work. In those cases where the drain breaks, the authors for the above technique recommend advancing a long slender hemostat along the drain tract with image intensification and grasping the retained drain [9].

Lazarides et al. [10] describe inserting the sharp end of an appropriately sized Steinmann pin within the drain lumen to cut the suture holding the drain in place. By inserting it within the lumen, the surrounding soft tissues are protected and the suture is cut from within the drain lumen.

Rue and Johnson [11] describe removing silicone drains, which are much softer than polyvinyl chloride (PVC) drains used in Hemovac drains, by grasping the tubing with a clamp. They apply gentle traction while twisting the drain five to seven times; with this technique, they were able to free the drain from the suture in 13/16 test cases in a porcine model.

In the cardiovascular literature, a technique was described for inserting an angioplasty balloon catheter along the drain tract and into the retained drain lumen under image intensification. The balloon is then inflated within the drain lumen creating an interference fit. The retained drain is then extracted by pulling out the angioplasty balloon catheter [12].

If the above techniques fail, the patient may have to return to the operating room to open the incision to retrieve the retained drain under general anesthesia.

### 2.4. Potential Complications

The general surgery literature includes a variety of complications with retained drains, including abdominal fistulas, abdominal abscesses, and intestinal obstruction [13–15]. In orthopedics, many drains are in joints and priority is placed on removal to reduce the risk of infection, cartilage damage, or restricting range of motion. There are no controlled studies on drain removal in orthopedics as they rarely occur. No reported adverse events were reported in the orthopedic literature related to those drains left in the wound after they broke. Gausden et al. [16] reviewed seven cases of retained drains after lumbar spine surgery over an 18-year period. Of these, five were removed surgically and two remained in situ with no complications at 2-year follow-up. Zeide and Robbins [4] described seven cases of retained drains, of which three were removed under conscious sedation and four were left in situ. The drains left in situ resulted in no complications at the time of publication. The authors reported that vinyl chloride, a product released while making the drain material polyvinyl chloride (PVC), has been linked to angiosarcoma in rat models and in PVC factory workers, but there is no evidence that this release occurs in relation to retained drains [4]. Therefore, there is little evidence in the literature to recommend for or against removal of retained drains unless a specific problem exists (i.e., range of motion limitations).

## 3. Conclusion

Though a rare event, retained surgical drains are a completely preventable complication and should not occur. They can inhibit recovery and create anxiety leading often to their removal under general anesthesia. By consistently employing one of the preventative techniques described, the incidence of this avoidable complication will diminish significantly.
